# Enhancing reading speed: the reading acceleration effect in Italian adult readers

**DOI:** 10.3389/fpsyg.2024.1394579

**Published:** 2024-07-31

**Authors:** Denisa Adina Zamfira, Giuseppe Di Dona, Martina Battista, Francesco De Benedetto, Luca Ronconi

**Affiliations:** ^1^School of Psychology, Vita-Salute San Raffaele University, Milan, Italy; ^2^Division of Neuroscience, IRCCS San Raffaele Scientific Institute, Milan, Italy; ^3^MoMiLab Research Unit, IMT School for Advanced Studies Lucca, Lucca, Italy

**Keywords:** reading acceleration program, visual attention (VA), visual perception, developmental dyslexia (DD), reading

## Abstract

**Introduction:**

Enhancing reading efficiency is of paramount importance in various academic, professional and clinical domains. Previous research, mostly from a single laboratory, has shown that externally imposed time constraints by means of text fading can enhance reading fluency in children and adults with varying reading abilities and in different languages.

**Methods:**

In the present study, we aimed at replicating and extending previous results in Italian readers. Three experiments (N = 90) were conducted: (i) to investigate the effects of continuous fading compared to character-wise fading, (ii) to investigate the influence of enlarged inter-letter spacing on reading acceleration outcomes, and (iii) to probe whether reading gains can be reliably observed off-line (after the acceleration) by comparing accelerated reading with an analog non-accelerated procedure.

**Results:**

Overall, results corroborate previous findings revealing that participants read 40% faster during the reading acceleration procedure, while maintaining the same accuracy levels. Continuous fading proved to be more effective than character-wise fading in enhancing reading speed, while larger inter-letter spacing did not significantly affect the reading speed gain. Albeit the non-clinical nature of our sample and its numerosity circumscribe the potential generalization, taking into account individual differences in the initial reading time, data suggests that reading acceleration leads to larger off-line speed increments with respect to non-accelerated reading.

**Discussion:**

Taken together, these findings offer valuable insights for the future application of reading acceleration procedures as part of multisession training programs for improving reading proficiency in a diverse range of clinical and non-clinical populations.

## 1 Introduction

Reading is a complex cognitive ability that has emerged recently in human evolutionary history. Nonetheless, it is a fundamental skill in modern society, with an increasing number of contemporary situations and professions, not only in the academic context, requiring the rapid and efficient processing of large amounts of written material. When reading a text, eye movements have to be sequentially deployed in order to land successively and quickly on each subsequent word, so as to bring it into high-acuity foveal vision (Laycock and Crewther, 2008). This process is highly dependent on visuo-spatial attention (Stolz and McCann, 2000; Vidyasagar and Pammer, 2010; Franceschini et al., 2012; Montani et al., 2014) and perception (Gibson, 1970; Wong and Gauthier, 2007; Zorzi et al., 2012; Gori and Facoetti, 2014; Liu et al., 2016), and any disruption in the detection, analysis, or identification of letters may result in slow and inaccurate reading. In addition, text comprehension requires word-to-text integration and is highly reliant on different aspects of attention and memory (McNamara and Magliano, 2009; Perfetti and Stafura, 2014).

Correct reading acquisition during early developmental stages is crucial to becoming proficient. First, the specific and culture-dependent rules used for representing speech as a sequence of visual symbols (grapheme-to-phoneme conversion) had to be learned (Ziegler and Goswami, 2005; Perfetti and Stafura, 2014). Afterwards, increasingly automatic decoding strategies are generated allowing attention and cognitive resources to be focused more on comprehension and understanding rather than struggling with visual decoding (LaBerge and Samuels, 1974; Nagler et al., 2016). However, in some cases, the acquisition of reading may falter, as well as the automatization of its core mechanisms, leading to slowness and difficulties, as in the case of poor and dyslexic readers (Gori and Facoetti, 2015). Other cognitive deficits, such as those derived from traumatic brain injuries or normal aging, can also affect decoding, fluency, and comprehension due to their impact on attention, working memory, and executive function processes indispensable for reading (Commodari and Guarnera, 2008; Kirova et al., 2015; Ratiu et al., 2022).

Breznitz and collaborators first showed that both impaired and unimpaired readers can enhance their reading speed and fluency, while maintaining high comprehension levels, if they are forced to read faster than their normal reading rate (Breznitz and Share, 1992; Breznitz, 2006). This effect is defined as the ‘reading acceleration effect’ (RAE) and can typically be observed in a three-condition within-subject design. In the first condition, participants are asked to read computerized reading stimuli (e.g., words, sentences, or short paragraphs) at their own pace (‘self-paced’ pre) while their reading time is recorded. Subsequently, during the ‘fast-paced’ condition, the text appears on the screen and, as the reader begins to read, it is progressively erased following the reading direction at the fastest rate recorded in the previous self-paced condition. In the last condition, participants are once again asked to read at their normal rhythms while no time constraints are imposed (‘self-paced’ post). During all conditions, participants are presented with questions to ensure that a high level of comprehension is maintained. In this single-session experimental design, participants typically tend to improve their reading speed in the fast-paced reading condition; however, less is known on whether these effects persist after the experimental manipulation in a single session. In previous investigations, it has been shown to revert back to baseline levels during the final self-paced condition, suggesting that such RAE is temporary in nature (Breznitz and Share, 1992; Korinth and Nagler, 2021). Several studies have subsequently confirmed that through externally-imposed time constraints it is possible to momentarily increase reading rates in children (Breznitz and Share, 1992; Breznitz, 1997a,b,c; Bar-Kochva and Hasselhorn, 2015) and adults (Breznitz et al., 1994), with different levels of reading abilities and in different languages (see Korinth and Nagler, 2021 for a recent review). For instance, adults reported an improvement in reading speed of 10–12% in fast-paced conditions as compared to self-paced ones (Breznitz et al., 1994), whereas children reported an enhancement of 15–30% (Breznitz and Share, 1992; Breznitz, 1997b). Moreover, a greater reduction in decoding errors, along with greater improvements in reading speed and comprehension, have been reported for dyslexic children in comparison with typically developing controls (Breznitz, 1997a,c), suggesting a relatively higher advantage for slow or impaired readers. While most studies involved Hebrew-speaking participants (Breznitz and Share, 1992; Breznitz, 1997a,b,c), reading acceleration effects have also been observed in English (Breznitz et al., 1994) and German (Bar-Kochva and Hasselhorn, 2015).

To investigate whether prolonged exposure to the reading acceleration procedure could lead to more strong or durable effects, Breznitz’s group developed a training divided into multiple sessions, the Reading Acceleration Program (RAP) (Breznitz, 2006; Breznitz et al., 2013). The RAP is implemented using the same paradigm as the RAE (i.e., self-paced pre, fast-paced, and self-paced post reading conditions), typically in a between-subject design, but rather than taking part in a single-session experiment, participants undergo the training several times on multiple days (i.e., from 9 to 60 training sessions over a period of 1 up to 3 months) (Breznitz, 2006; Snellings et al., 2009; Paige, 2011; Breznitz et al., 2013; Horowitz-Kraus et al., 2014a,b; Horowitz-Kraus and Holland, 2015; Nagler et al., 2015; Dai et al., 2016; Horowitz-Kraus, 2016; Korinth et al., 2016; López-Escribano, 2016; Nevo et al., 2016; Franceschini et al., 2017). Most RAP studies reported increased reading speed and increased or preserved comprehension levels in post training paper-based reading evaluations (Korinth and Nagler, 2021), and in some cases beneficial effects persisted for up to 6 months after the end of the training (Breznitz et al., 2013). Consistent with prior research on the RAE, the RAP led to significant improvements in reading speed during fast-paced conditions. Interestingly, repeated application of the fading procedure increased reading speed also in self-paced conditions recorded after each session across the whole training (Nagler et al., 2015; Korinth et al., 2016). RAP studies have extended previous single-session evidence to Chinese (Dai et al., 2016), Italian (Franceschini et al., 2017), Spanish (López-Escribano, 2016), and Dutch (Snellings et al., 2009) readers, suggesting that reading acceleration is a highly generalisable phenomenon, independent of reading direction, orthographic transparency of the language, or visual complexity of graphemic representations.

Although there is an increasing clinical interest in reading acceleration interventions in individuals with reading difficulties, experimental investigations of the RAE in non-impaired adult populations are still scarce (Breznitz et al., 1994), and is not possible to exclude developmental influences on previously reported RAE and RAP outcomes. Investigating single-session reading acceleration effects in adult typical readers is *per se* extremely important to better understand the methodological, perceptual, cognitive, and psycholinguistic variables affecting reading acceleration, as well as its neurophysiological correlates. All these aspects are crucial to design more effective reading trainings, which would potentially benefit not only poor and dyslexic readers, but also individuals with acquired brain damage (Tabet et al., 2020; Pei and O’Brien, 2021; Ratiu et al., 2022) or aging-related reading difficulties (Stine-Morrow et al., 1996; Rayner et al., 2006b; De Luca et al., 2017; Liu et al., 2017; Warrington et al., 2018).

Notably, there are almost no independent replications of the studies made by Breznitz and her research group. Almost all of the studies that employed the paradigm eliciting the reading acceleration effect were carried out by either Breznitz or her collaborators. Furthermore, even studies not directly conducted by Breznitz’s group still had their indirect involvement (Korinth and Nagler, 2021). Recently, Korinth and Nagler (2021) pointed out that only three studies investigating reading acceleration were truly independent from Breznitz’s work (Paige, 2011; Dai et al., 2016; Franceschini et al., 2017; Korinth and Nagler, 2021). However, they used variations of the original paradigm, as their main objective was not to explicitly assess the effect of the acceleration phenomenon or program on reading performance. Specifically, Paige (2011) combined the RAP with the currently most accepted intervention for ameliorating fluency (i.e., repeated reading; see Lee and Yoon, 2017) and compared text presented at an accelerated rate to the traditional still-text method in English-speaking children with reading disorders (Paige, 2011). This study differs from classical research on reading acceleration because the same reading material was used during the different training sessions. Results indicate that both the static and fast-paced approaches performed equally, with only moderate effects on oral reading fluency (Paige, 2011). However, using the same reading stimuli in each session may have greatly impacted such results. Another independent study was conducted by Franceschini et al. (2017), who implemented a visuo-attentional computer game based on Breznitz’s research work, called “The Library Tower,” and used it following the RAP training approach in Italian children with dyslexia (Franceschini et al., 2017). Their aim was to test the efficacy of different visuo-attentional trainings in restoring efficient global before local visual processing. The authors reported that both an action videogame training and the RAP-based training were able to improve reading skills (Franceschini et al., 2017), confirming that such procedure appears to be feasible also in the Italian language and paving the way for potential clinical applications both in child and adult impaired readers. Lastly, Dai et al. (2016) compared three groups of Chinese dyslexic children performing 9 training sessions during non-accelerated reading, character-wise fading reading, or word-wise fading reading (Dai et al., 2016). Compared to non-accelerated reading, character-wise accelerated training improved comprehension in post-training paper-based reading assessments, while word-wise acceleration enhanced reading speed (Dai et al., 2016).

To our knowledge, only one previous study has investigated the RAE in a transparent alphabetical language, i.e., German, using different fading techniques. In particular, children with and without reading disabilities were tested using letter-by-letter fading, word-wise fading, and orthographic unit (i.e., prefix, suffix, stem) fading (Bar-Kochva and Hasselhorn, 2015). This comparison revealed that, irrespective of the orthographic unit manipulated, externally-imposed time constraints exerted a positive influence on reading performance (Bar-Kochva and Hasselhorn, 2015). Therefore, the examination of different fading manipulations and their impact on RAP and RAE outcomes is still a largely neglected field.

The present study aims to implement and test a tool capable of accelerating reading in a single session design, a step which is important for the future application of RAP training to adult clinical populations with reading difficulties. Considering the current replication crisis in psychological sciences (Aarts, 2015), to enhance the validity of the RAE and RAP approaches in ameliorating reading it is important to conduct independent replications of the key findings previously reported. Moreover, while considerable research has been conducted on the neurocognitive processes involved in the reading acceleration effect and on its clinical applications (Korinth and Nagler, 2021), only few studies have delved into methodological aspects of this ameliorative technique (Bar-Kochva and Hasselhorn, 2015; Dai et al., 2016). Thus, based on these premises and the evidence reviewed above, we run three distinct RAE experiments on Italian adult readers with the following aims:

- to create a database of Italian sentences, with corresponding questions and answers, that can be used to implement and test the reading acceleration phenomenon and training; to our knowledge no such database exists at present;
- to replicate Breznitz’s findings, in a completely independent way and using a paradigm as similar as possible to the original, on a sample of competent adult readers;
- to explore whether different types of text fading procedures could have an impact on the effects yielded by the acceleration phenomenon. The stimuli could in fact either fade in a letter-by-letter fashion or, alternatively, be progressively covered by a sliding occlusor. With the latter we aimed to reach a faster fading rate;
- to investigate whether inter-letter spacing can modulate the RAE; this question is stimulated by several works (Spinelli et al., 2002; Perea et al., 2012, 2016; Zorzi et al., 2012; Scaltritti et al., 2019) showing that larger inter-letter spacing is effective in improving reading performance by reducing the detrimental effect of visual crowding on letter/word identification and recognition.
- to control for the effect of mere reading exposure involved in the RAE by comparing the reading gain observed during the accelerated procedure with that obtained by reading the same amount of material without fading (non-accelerated procedure).

## 2 Materials and methods

### 2.1 Participants

A total of 90 healthy adults (*F* = 45, Mean age = 23.10, SD = 3.20) with normal or corrected to normal vision and normal hearing were recruited from the student population of the Vita-Salute San Raffaele University. Each experiment included 30 participants with equal distribution of sex (*F* = 15, *M* = 15), similar mean age (Exp. 1: 23.15, Exp. 2: 23.65, Exp. 3: 22.45) and a comparable number of years of education (Exp. 1: 16.60, Exp. 2: 16.25, Exp. 3: 16.20). None of the participants took part in more than one of the experiments. All participants were Italian native-speakers with varying levels of reading abilities within the normal range, and no history of reading, neurological, psychiatric disorders or other medical conditions. They received neither payment nor credits to participate in the study. Prior to the beginning of the experiments, participants were instructed about the experimental procedure and agreed to participate by signing an informed consent document. The study protocol was approved by the Ethical Committee of San Raffaele Hospital and was conducted according to the policies and ethical principles of the Declaration of Helsinki.

### 2.2 Stimuli

The majority of studies investigating reading acceleration effects have used single sentences or short passages (i.e., 1 to 10 declarative sentences) with mainly high-frequency words (Breznitz and Share, 1992; Breznitz et al., 1994; Breznitz, 1997a,b,c, 2006; Bar-Kochva and Hasselhorn, 2015). However, according to Hiebert and Fisher, (2007), at least three reading material characteristics may influence reading performance: word length, sentence length, and word frequency. Words with a greater number of syllables require longer processing times, whereas shorter words are decoded and understood more easily (McNerney et al., 2011). Sentence length affects reading comprehension in a similar way (Goldman et al., 1980). Moreover, word frequency is thought to affect reading performance by acting on lexical access, i.e., the retrieval of concepts from the mental lexicon, with highly frequent words facilitating efficient processing and infrequent words requiring more cognitive effort for recognition (Nagler et al., 2014, 2016). Hence, it is essential to explore whether comparable reading speed improvements can be observed when reading sentences with varying lengths (i.e., short, medium, and long) and containing words with different frequency ranges (i.e., from commonly used to more unfamiliar words), or if the previous RAE and RAP outcomes were primarily influenced by the presence of short sentences with mainly high-frequency words.

Given the lack of Italian databases of stimuli adequately controlled from the point of view of psycholinguistic aspects (e.g., word frequency and length, sentence cumulative frequency and length, target position in the sentence), we conceptualized and created a database of 275 sentences with related multiple-choice comprehension questions and pertinent answers.^1^ Sentences were created, using newspapers, novels, textbooks, blogs, and specialist magazines. The text had different lengths and covered various topics, including general culture, science, literature, as well as everyday-life situations. Word frequency (word per million, wpm) was computed from the SUBTLEX-IT corpus (Crepaldi et al., 2015), and then transformed into Zipf frequency by applying log_10_ to wpm frequency (Van Heuven et al., 2014; see Supplementary materials). Frequency of articles, prepositions and punctuation was not computed to avoid a bias in sentence frequency. Each sentence included a subject, a verb, a direct object, one or more modifiers, and subordinating or coordinating conjunctions. Sentences could have either an active or passive voice. The comprehension questions were designed so that the sentences had to be completely read in order to be correctly answered, requiring either the correct understanding of the relationship between words or the recall of specific individual words contained in the sentences. In order to balance possible primacy and recency memory effects, questions referred to target words positioned along different points in the sentence. Target position within each sentence was computed by dividing the position of the target word (e.g., 5 if the target is the 5^th^ word in the sentence) by the total number of words thus obtaining a proportion. Based on such proportions, different target position classes were assigned: initial (0–25%), initial-mid (25–50%) mid (50–75%), mid-final (75–99%) final (100%). Among the four plausible alternative answers, one was correct, two were clearly wrong and one served as a distractor as it was created to be phonologically, semantically, or visually similar to the correct answer (e.g., “Pane” - *Bread* -, “Cane,” *Dog,* an appropriate example in English would be *Cloud* and *Aloud*), so as to make answering more challenging (see Supplementary materials). The same database of stimuli was used for each experiment.

### 2.3 General procedure

At the beginning of each experiment, participants filled out a computer-based questionnaire assessing their demographics (e.g., age, sex, years of education). Then, they were asked to perform a computerized reading task in which pseudo-randomly selected sentences appeared one at a time on the display followed by comprehension questions with four multiple-choice answers. The reading task was programmed and implemented through PsychoPy - Psychology software in Python (Peirce, 2007). Text stimuli were displayed in black with “Consolas” monospaced font at 25 pt (letter width ~ 3 mm) over a gray background with 60% contrast on a “28 × 21 cm 15” LCD laptop screen (1,280 × 720 pixels; 60 Hz refresh rate) situated 60 cm in front of participants. Shorter sentences were presented on a single line, while longer items were presented on two lines of text with 3 mm vertical spacing between them.

**Figure 1:**
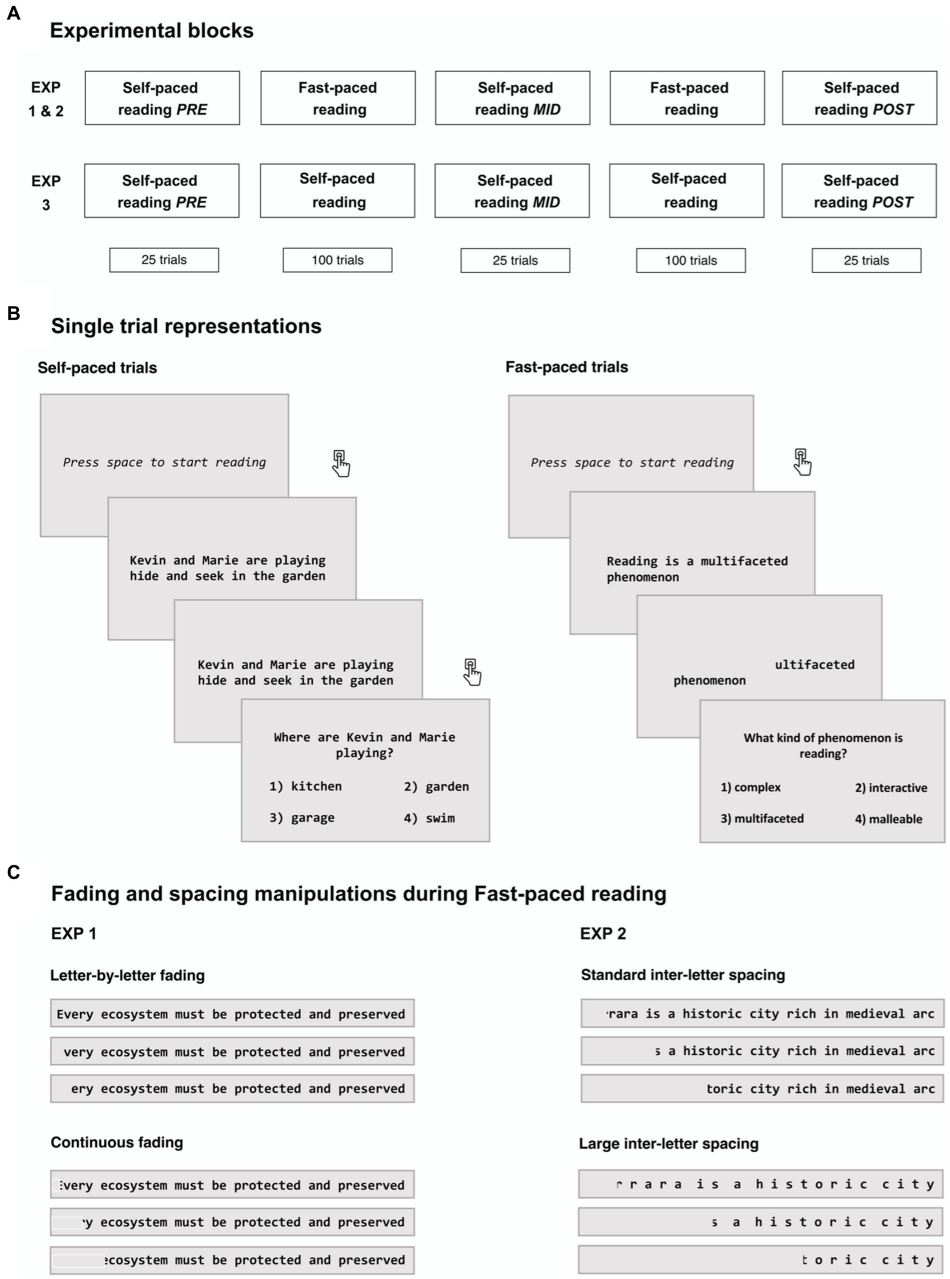
Schematic overview of the experimental design and manipulations. In both Experiment 1 and 2 the first, third, and fifth blocks consisted in self-paced reading, while the second and the fourth block consisted in fast-paced reading. In Experiment 3, the same blocks division was used, but fast-paced trials were substituted with still-text presentation **(A)**. During self-paced trials participants were asked to press the spacebar key to start reading sentences and as soon as they were finished. During fast-paced reading trials, after participants pressed the spacebar for the appearance of each individual sentence, letters began to progressively disappear from the screen from left to right. Comprehension was ensured after each trial with multiple-choice questions **(B)**. During fast-paced reading, in Experiment 1 we compared letter-by-letter fading, in which letters were faded one by one from left to right, with continuous fading, in which sentences were progressively covered by a sliding rectangular occlusor, while in Experiment 2 we compared standard and large (double) inter-letter spacing **(C)**.

For Experiments 1 and 2, the task was divided into two main experimental conditions, namely self-paced reading and fast-paced reading (see below), that followed each other during five blocks. Specifically, the first, third, and fifth blocks consisted in self-paced reading, while the second and the fourth block consisted in fast-paced reading. For Experiment 3 participants performed five blocks of self-paced reading (see Figure 1A). Prior to the beginning of each block, participants received specific on-screen instructions.

#### 2.3.1 Self-paced reading

During self-paced reading, participants were instructed to silently read the sentences at their own pace, and then answer the related multiple-choice questions. In more detail, at the beginning of each trial, participants were asked to press the spacebar on the keyboard positioned in front of them as soon as they were ready to start. Subsequently, sentences appeared one at a time at the center of the computer screen. Participants were instructed to read the sentence immediately upon its appearance and press the spacebar key as soon as they were finished reading. Then, a comprehension question related to the previously presented sentence appeared, along with 4 possible multiple-choice answers (numbered from 1 to 4), of which only one was correct. Participants were asked to answer by pressing the 1–4 keys, with no time constraints. After responding, they were prompted by on-screen instructions to press the spacebar key as soon as they were ready to start the subsequent trial (see Figure 1B).

Per-letter reading time was calculated for each correctly-answered sentence as the time (in *s*) elapsed between when participants pressed the spacebar for the sentence presentation and when they pressed it because they had finished reading, divided by the number of letters. For Experiments 1 and 2, these values were used as the initial fading rate in the following fast-paced condition, which was different for each participant and subsequently adjusted based on their performance in the comprehension questions (see *Fast-paced reading* section). Each participant completed 75 self-paced trials during three separate blocks, interleaved by two blocks of 100 fast-paced trials. In Experiment 3 participants completed the same three blocks of self-paced trials (25 trials each) interleaved by two longer blocks of 100 self-paced trials.

#### 2.3.2 Fast-paced reading

During fast-paced reading conditions (Experiments 1 and 2), after participants pressed the space bar for the appearance of each individual sentence, letters began to progressively disappear from the screen following reading direction (i.e., from left to right, see Figure 1B). As above mentioned, the initial fading speed was calculated for each participant using their average reading time on correctly answered questions in the previous self-paced phase. After the first 10 trials, the letter disappearing rate changed according to the participant’s comprehension accuracy. More in detail, the performance of 10 consecutive trials was taken into account: when the participant correctly answered 10 out of 10 trials (accuracy = 100%) the disappearing rate increased by 4 ms, it was maintained constant when accuracy was between 80 and 100%, and decreased by 4 ms with an accuracy lower than 80%.

#### 2.3.3 Manipulation of text fading: letter-by-letter vs. continuous (experiment 1)

In the first experiment, we aimed to independently replicate Breznitz’s findings on a sample of adult unimpaired readers using a paradigm as similar as possible to the original. Moreover, we investigated whether different types of text fading procedures could have an impact on the outcomes yielded by the RAE. To this aim, we manipulated the method through which sentences disappeared from the screen in two different fast-paced blocks. More specifically, in one of the two blocks letters were faded in a character-wise manner (i.e., letters were erased one by one from left to right), while in the other block sentences were progressively covered by a sliding rectangular occlusor (continuous fading). In the latter case, the speed at which the occluder covered the letters was dependent on the refresh rate of the screen, and it could happen that more than one letter was covered at the same time, or that one letter was only partially covered. The occlusor had the same color of the background, making it impossible for participants to notice the different disappearing methods between the two blocks (see Figure 1C). In each of the two blocks, presented in counterbalanced order, participants completed 100 fast-paced trials.

#### 2.3.4 Manipulation of inter-letter spacing: standard vs. large (experiment 2)

In the second experiment, we aimed to investigate whether increasing the space between characters (i.e., reducing visual crowding) affected the beneficial effects of RAE. During fast-paced conditions, in this case implemented using continuous fading, two distinct types of inter-letter spacing were used: Standard, where the space between each subsequent letter was set as standard of the used font (i.e., Consolas), and Large, where the space between each subsequent letter was doubled (see Figure 1C). In each of the two blocks, presented in counterbalanced order, participants completed 100 fast-paced trials.

#### 2.3.5 Control experiment: still-text method (experiment 3)

Given that in the absence of a control group it would be difficult to interpret any change in reading speed during and/or after the experimental manipulations, in a third experiment, we specifically isolated the contribution of reading practice *per se* in the absence of forced acceleration. Indeed, it is possible that the decrease in reading time may emerge also without acceleration. Instead of presenting fast-paced trials, we only presented self-paced trials in shorter and longer blocks to adhere to the structure of the previous experiments. In this way, also in Experiment 3 each participant completed three blocks of self-paced trials (Pre, Mid and Post; 25 trials each) interleaved by two longer blocks of 100 self-paced trials.

### 2.4 Statistical analysis

All the statistical analyses were conducted using R (R Core Team, 2013) with customized scripts. The ANOVAs were performed using the ‘ez’ package while post-hoc comparisons were conducted using paired-sample t-tests included in the base ‘stats’ package. When the assumption of sphericity was violated, we applied the Greenhouse–Geisser correction. To control for multiple comparisons, the False-Discovery Rate correction (FDR) was applied.

#### 2.4.1 Experiment 1 data analysis

Two repeated-measures Analysis of Variance (rm-ANOVAs) were performed on letter reading time and accuracy recorded during self-paced conditions. In the first ANOVA we considered Session (3 levels: Pre, Mid, and Post) as within-subject factor, and Block Order (Letter-by-letter Fading – Continuous Fading, Continuous Fading – Letter-by-Letter Fading) as a between-subject factor. This first analysis allowed us to evaluate the time course of the effect, and also to control whether the order of the different types of inter-letter spacing employed had a different impact on such time course. In the second ANOVA we considered Session (Pre, Post) and Condition (Letter-by-letter Fading, Continuous Fading) as within-subject factors to understand if the different fading techniques yielded differential transfer effects in terms of reading speed enhancement. Note that in this analysis, the “Pre” and “Post” sessions refer to single condition blocks thus they may correspond to different levels of the factor Session (Pre, Mid, Post) used in the first ANOVA depending on the Block Order. These two analyses on letter reading time were performed only for trials in which participants answered correctly to the comprehension questions.

Furthermore, to compare online effects of the different text fading techniques, a third ANOVA was performed on letter disappearing rate and accuracy on all trials recorded during fast-paced conditions with Condition (Letter-by-letter Fading, Continuous Fading) as within factor and Block Order (Letter-by-Letter Fading – Continuous Fading, Continuous Fading – Letter-by-Letter Fading) as between factor.

Finally, to investigate differences in reading performance between self-and fast-paced reading a t-test was performed to compare the letter reading time recorded during the self-paced reading averaged across sessions with the letter disappearing rate (only for correct trials) averaged across fast-paced conditions.

#### 2.4.2 Experiment 2 data analysis

Similarly to Experiment 1, two rm-ANOVAs were performed on letter reading time and accuracy recorded during self-paced conditions. In the first ANOVA we considered the Session (3 levels: Pre, Mid, and Post) as within-subject factor, and Block Order (Large Interletter Space – Standard Interletter Space, Standard Interletter Space – Large Interletter Space) as a between-subject factor. This first analysis allowed us to evaluate the time course of the effect, and also to control whether the order of the different types of inter-letter spacing employed had a different impact on such time course. In the second ANOVA we considered Session (Pre vs. Post) and Interletter spacing (Standard, Large) as within-subject factors to understand if different spacing levels yielded differential transfer effects in terms of reading speed enhancement. In this analysis, the “Pre” and “Post” sessions refer to single condition blocks thus they may correspond to different levels of the factor Session (Pre, Mid, Post) used in the first ANOVA depending on the Block Order. These two analyses on letter reading time were performed only for correctly answered trials.

Furthermore, to compare online effects of different inter-letter spacing on letter disappearing rate, a third ANOVA was performed on letter reading time and accuracy on all trials recorded during fast-paced conditions with Condition (Standard Interletter Space, Large Interletter Space) as within factor and Block Order (Large Interletter Space – Standard Interletter Space, Standard Interletter Space – Large Interletter Space) as between factor.

Finally, to investigate differences in reading performance between self-and fast-paced reading a t-test was performed to compare letter reading time recorded during the self-paced condition averaged across sessions with the disappearing rate (only for correctly answered trials) averaged across fast-paced conditions.

#### 2.4.3 Experiment 3 data analysis

We performed two rm-ANOVAs on letter reading time and accuracy recorded during the shorter self-paced reading conditions. In this case we only considered the factor Session (3 levels: Pre, Mid, and Post) as within-subject factor (note that we did not include Block Order as a factor as the two longer self-paced conditions were identical). Post-hoc t-tests were performed to understand possible differences between all factor levels. As for the previous experiments, these analyses were performed on trials in which participants answered correctly to the multiple-choice comprehension questions. Secondly, we performed two paired-sample t-tests to compare the differences in letter reading time and accuracy between the two longer self-paced blocks.

#### 2.4.4 Correlation analyses

First, the letter reading time recorded at the first self-paced session (pre) in all experiments was extracted. Second, the Δ letter reading time was computed by subtracting the letter reading time recorded after (self-paced post) from the one recorded before (self-paced pre) the two fast-paced conditions in Exp. 1 & 2 and before and after the longer self-paced blocks in Exp. 3. Finally, a correlation analysis was performed between the initial letter reading time and the Δ letter reading time to evaluate the relationship between baseline reading speed levels and the magnitude of the RAE.

#### 2.4.5 Omnibus analysis with linear mixed effects models

As a last analysis, we used the combined single-trial data from all the self-paced conditions of all the 3 experiments to explore possible differences in terms of reading time decrement across experiments, while also capturing the variability stemming from individual differences in initial reading times that may have induced differentiated results across experiments.

We fitted the linear mixed effects model using the package ‘lmerTest’ (Kuznetsova et al., 2017), in R (R Core Team, 2013) with the formula “Letter reading time ~ Session * Experiment * Initial Reading Time + (1|participant) + (1|item)” which included Session (Pre, Mid, Post), Experiment (Exp. 1, Exp. 2, Exp. 3) as categorical predictors, Initial Reading Time as continuous predictor and all the possible interactions between the three. Participant and Item (i.e., sentence) were included as random factors. An ANOVA (F-test) was operated on the model to explore possible effects and interactions. Post-hoc tests were performed on the model’s estimated marginal means (EMMs) with the package ‘emmeans’ (Lenth et al., 2018) using the FDR adjustment for the *p*-value when multiple comparisons were performed. Note that the inclusion of a continuous predictor in the interaction with other categorical ones results in the estimation of EMMs for the other categorical factors considering the mean value of the continuous one. In this specific case, the EMMs are estimated for the mean Initial Reading Time across all of the experiments thus representing an “average reader.”

## 3 Results

### 3.1 Experiment 1

#### 3.1.1 Reading time

The first ANOVA on self-paced letter reading time with Session (Pre, Mid, Post) as within factor and Block Order (Letter-by-letter Fading – Continuous Fading, Continuous Fading – Letter-by-Letter Fading) as between factor showed a main effect of Session *F* (1.37, 38.56) = 20.74, *p* < 0.001, η2 = 0.12. Post-hoc tests showed that letter reading time diminished significantly from the Pre session (*M* = 66.30 ms SE = 3.21 ms) to the Mid-session (*M* = 56.90 ms SE = 2.66 ms) *t* (29) = 4.13, p_FDR_ < 0.001, from the Mid to the Post session (*M* = 53.30 ms SE = 2.39 ms) *t* (29) = 2.96, p_FDR_ = 0.005, and from the Pre to the Post one *t* (29) = 12.94, p_FDR_ < 0.001. No other effect reached significance (all ps > 0.29).

The second ANOVA including Session (Pre, Post) and Condition (Letter-by-letter Fading, Continuous Fading) showed a main effect of Session *F* (1,29) = 26.03, *p* < 0.001, η2 = 0.04. Post-hoc tests showed that letter reading time diminished significantly from Pre (*M* = 61.60 ms SE = 2.71 ms) to Post (*M* = 55.10 ms SE = 2.45 ms) *t* (29) = 5.16, p_FDR_ < 0.001. No other effect reached significance (all ps > 0.39).

The third ANOVA on letter disappearing rates during fast-paced reading with Condition (Letter-by-letter Fading, Continuous Fading) as within factor and Block Order (Letter-by-Letter Fading – Continuous Fading, Continuous Fading – Letter-by-Letter Fading) as between factor showed a main effect of Condition *F*(1,28) = 13.92, *p* < 0.001, η2 = 0.13. Post-hoc t-test showed a shorter mean letter fading time in the Continuous Fading condition (*M* = 27.00 ms, SE = 1.21 ms) with respect to the Letter-by-Letter Fading condition (*M* = 38.70 ms, SE = 3.58 ms) *t* (29) = 11.37, p_FDR_ = 0.001. No other effect reached significance (all ps > 0.14).

The t-test comparing performance during self-paced trials, averaged across sessions, with that recorded during the fast-paced trials showed that participants’ fast-paced letter disappearing rate (*M* = 32.90 ms, SE = 2.21) was faster as compared to their self-paced letter reading time (*M* = 58.8 ms, SE = 2.49) *t* (29) = 9.83, p_FDR_ < 0.001.

#### 3.1.2 Accuracy

The first ANOVA on accuracy did not show significant effects (all ps > 0.056). The second ANOVA on accuracy showed a main effect of Session *F* (1,29) = 4.25, *p* = 0.048, η2 = 0.02. Post-hoc t-test comparing the accuracy recorded in the “self-pre” (*M* = 0.94, SE = 0.004) as compared to the “self-post” session (*M* = 0.93, SE = 0.006) did not yield significant differences (p_FDR_ = 0.051). The ANOVA on accuracy for the fast-paced condition showed a main effect of Condition *F* (1,28) = 26.12, *p* < 0.001, η2 = 0.29. Post-hoc tests showed that the Continuous Fading condition (*M* = 0.88, SE = 0.002), was slightly more challenging than the Letter-by-letter one (*M* = 0.91, SE = 0.005), *t* (29) = 5.08, p_FDR_ < 0.001.

**Figure 2:**
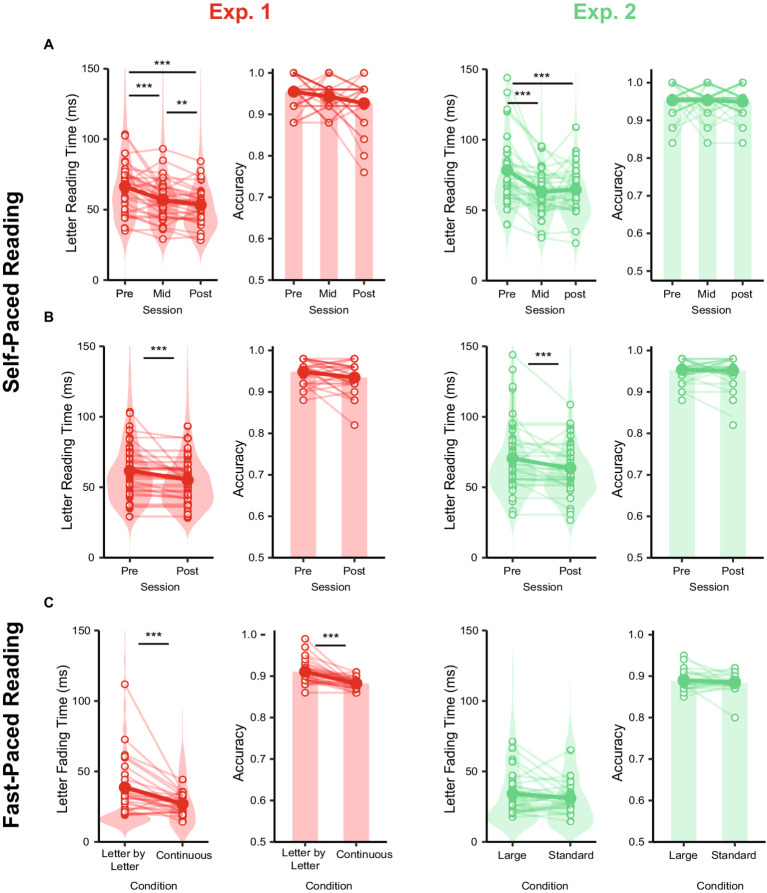
Results of Experiment 1 and 2. Letter reading time and accuracy recorded during self-paced-reading across sessions (Pre, Mid, Post) analyzed in the first ANOVA (Session x Block Order) for experiment 1 (red) and 2 (green) **(A)**. Letter reading time and accuracy recorded during self-paced-reading across sessions (pre, post) analyzed in the second ANOVA (Session x Condition) **(B)**. Letter Fading time and accuracy recorded during fast-paced reading across conditions analyzed in the ANOVA (Session x Condition) **(C)**.

To summarize, these results indicate that participants exhibited a higher reading speed during the reading acceleration conditions as compared to the self-paced conditions while maintaining high levels of accuracy. Moreover, the continuous fading method resulted in a more pronounced increase in reading speed during the experimental manipulation as compared to the letter-by-letter fading method. In terms of transfer (i.e., offline) effects on self-paced reading after the acceleration, both methods yielded similar effects (See Table 1 for a summary of the main results) (Figure 2).

**Table 1:** Summary of results from experiments 1, 2, and 3.

Results summary of experiments 1, 2 and 3						
Descriptive statistics: self-paced reading						
	Exp 1		Exp 2		Exp3	
Session	Letter reading time	Accuracy	Letter reading time	Accuracy	Letter reading time	Accuracy
Pre	66.3 (17.60)	0.95 (0.04)	78.3 (24.60)	0.95 (0.04)	85.5 (24.60)	0.94 (0.03)
Mid	56.9 (14.60)	0.94 (0.03)	62.9 (15.60)	0.95 (0.03)	71.8 (21.90)	0.95 (0.03)
Post	53.3 (13.10)	0.92 (0.05)	64.5 (17.10)	0.94 (0.03)	71.3 (23.60)	0.94 (0.05)

Inferential statistics: self-paced reading						
Test	Letter reading time	Accuracy	Letter reading time	Accuracy	Letter reading time	Accuracy
Pre vs. Mid	*t* (29) = 4.13***	/	*t* (29) = 15.38***	/	*t* (29) = 13.65**	/
Mid vs. Post	*t* (29) = 3.58**	/	/	/	/	/
Pre vs. Post	*t* (29) = 12.94***	/	*t* (29) = 13.74**	/	*t* (29) = 14.12***	/

Descriptive statistics: fast-paced reading (Exp 1 and 2) and self-paced reading (Exp 3)								
Fast-paced			Fast-paced			Self-paced		
Condition	Letter fading time	Accuracy	Condition	Letter fading time	Accuracy	Condition	Letter reading time	Accuracy
Letter-by-letter fading	38.70 (19.60)	0.91 (0.02)	Large interletter spacing	34.30 (14.00)	0.89 (0.02)	Self-paced (Block 1)	76.80 (21.00)	0.96 (0.01)
Continuous fading	27.00 (6.62)	0.88 (0.04)	Normal interletter spacing	31.10 (9.75)	0.88 (0.02)	Self-paced (Block 2)	77.40 (23.50)	0.96 (0.0.2)

Inferential statistics: fast-paced reading (Exp 1 and 2) and self-paced reading (Exp 3)								
Tests	Letter fading time	Accuracy	Test	Letter fading time	Accuracy	Test	Letter reading time	Accuracy
Letter by letter vs. continuous	*t* (29) = 11.72***	*t* (29) = 5.08***	Large vs. normal interletter	/	/	Self-paced (Block 1) vs. self-paced (Block 2)	/	/

### 3.2 Experiment 2

#### 3.2.1 Reading time

The first ANOVA including Session (Pre, Mid, Post) as within factor and Block Order (Large Interletter Space – Standard Interletter Space, Standard Interletter Space – Large Interletter Space) as between factor showed a main effect of Session *F* (1.72, 48.16) = 14.62, *p* < 0.001, η2 = 0.12. Post-hoc tests showed that letter reading time diminished significantly from the Pre session (*M* = 78.30 ms, SE = 4.49 ms) to the Mid-session (*M* = 62.90 ms, SE = 2.86 ms) *t* (29) = 15.38, p_FDR_ < 0.001, and from the Pre to the Post one (*M* = 64.50 ms, SE = 3.12 ms) *t* (29) = 13.74, p_FDR_ = 0.001. No other effect reached significance (all ps > 0.58). The second ANOVA including Session (Pre, Post) and Condition (Standard Interletter Space, Large Interletter Space) showed a main effect of Session *F* (1,29) = 14.41, *p* < 0.001, η2 = 0.03. Post-hoc tests showed that letter reading time diminished significantly from Pre (*M* = 70.50 ms, SE = 3.43 ms) to Post (*M* = 63.60 ms, SE = 2.69 ms) *t* (29) = 3.83, p_FDR_ < 0.001. No other effect reached significance (all ps > 0.67).

The third ANOVA on letter disappearing rate during fast-paced reading with Condition (Standard Interletter Space, Large Interletter Space) as within factor and Block Order (Large Interletter Space – Standard Interletter Space, Standard Interletter Space – Large Interletter Space) as between factor did not show any significant effect (all ps >0.18).

The t-test comparing performance during self-paced trials, averaged across sessions, with that recorded during fast-paced trials showed that participants’ fast-paced letter disappearing rate (*M* = 33.83, SE = 0.002) was faster with respect to their self-paced letter reading time (*M* = 68.46, SE = 0.003) *t* (29) = 16.03, p_FDR_ < 0.001.

#### 3.2.2 Accuracy

Neither the first nor the second ANOVAs on accuracy during the self-paced blocks showed significant effects (all ps >. 36). The ANOVA on accuracy during the fast-paced blocks did not yield any significant result (all ps >. 32).

To summarize, these results indicate that participants read faster during the reading acceleration conditions than during the self-paced conditions while maintaining similar levels of accuracy. No significant differences in reading speed were observed between the normal and large interletter spacing conditions during the experimental manipulation. In addition, in terms of transfer (i.e., offline) effects, participants showed higher reading speed during self-paced reading after the reading acceleration blocks, regardless of spacing (See Table 1 for a summary of the main results; Figure 2).

### 3.3 Experiment 3

#### 3.3.1 Reading time

The first ANOVA including Session (Pre, Mid, Post) as within subjects factor showed a main effect of Session *F* (1.54, 44.66) = 7.85, *p* = 0.002, η2 = 0.075. Post-hoc tests showed that letter reading time diminished significantly from the Pre session (*M* = 85.5 ms, SE = 4.49 ms) to the Mid-session (*M* = 71.8 ms, SE = 3.99 ms) *t* (29) = 3.74, p_FDR_ = 0.002, but not from the Mid to the Post session (*M* = 71.3 ms, SE = 4.32 ms) *t* (29) = 0.14, p_FDR_ = 0.89. There was also a significant increment in reading speed from the Pre to the Post session *t* (29) = 2.81, p_FDR_ = 0.013.

The t-test comparing performance during longer self-paced blocks showed that participants’ letter reading time did not change significantly (first block: *M* = 76.90, SE = 4.22; second block: *M* = 77.2, SE = 3.92) *t* (29) = 0.016, *p* > 0.99.

#### 3.3.2 Accuracy

The ANOVA on accuracy during the self-paced blocks did not show significant effects (*p* = 0.75). The paired sample t-test on accuracy during the longer self-paced blocks did not show any significant effect (*p* = 0.65).

To summarize, these results showed a decrease in self-paced reading between the Pre and the Mid-session but not between the Mid and the Post ones. No significant differences emerged comparing reading time in longer self-paced blocks that participants performed as an alternative to accelerated reading (See Table 1 for a summary of the main results).

### 3.4 Correlation analyses

**Figure 3:**
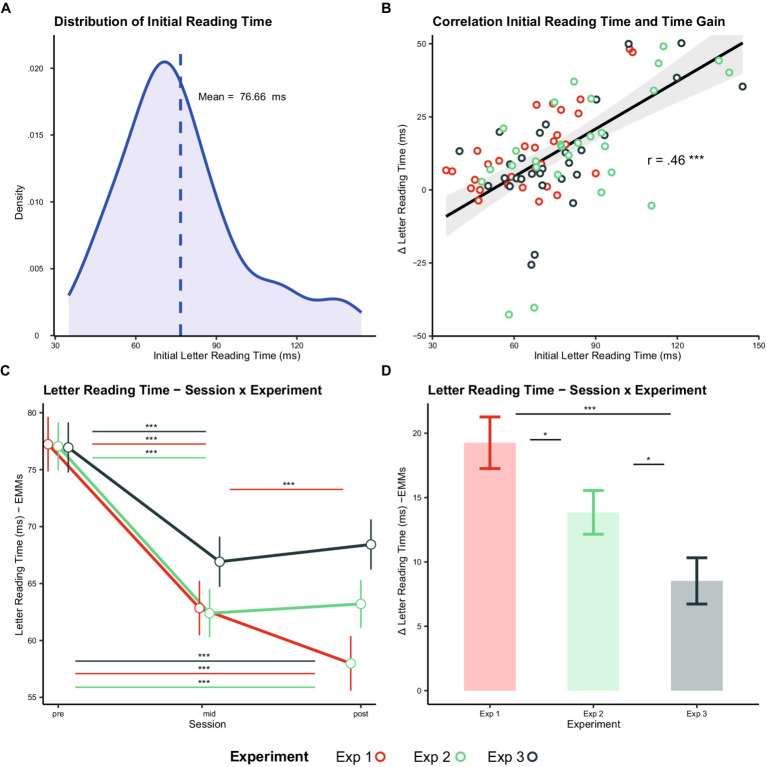
Results of correlation and comparison of the three experiments. Distribution of the initial reading time for all participants taking part in the 3 experiments **(A)**. Correlation between the magnitude of reduction of letter reading time (y-axis) and the initial reading time (x-axis) performed on combined data of all experiments. Data points represent individual observations from experiment 1 (red), 2 (green), and 3 (black) **(B)**. Self-paced reading time estimated with Linear Mixed Models for experiment 1 (red), 2 (green) and 3 (black) in the Pre, Mid and Post assessments performed throughout reading acceleration (Exp. 1 and 2) or non-accelerated control (Exp. 3) procedure **(C)**. Improvement in self-paced reading speed observed before vs. after the accelerated reading (or non-accelerated reading) procedure in the experiments as estimated with Linear Mixed Models **(D)**.

The correlation analysis showed a positive correlation between initial letter reading time and the reduction in letter reading time after the acceleration procedure *t* (88) = 4.86, r = 0.46, *p* < 0.001 (see Figures 3A,B), suggesting that the higher the initial letter reading time (i.e., the slower the reading performance), the higher the letter reading time reduction (i.e., the higher the reading speed gain).

### 3.5 Omnibus analysis

The ANOVA performed on the Linear Mixed Effects Model showed an interaction between Session and Initial Reading Time *F*(2, 6251.9) = 91.05, *p* < 0.001. Post-hoc tests showed that the slope of the Initial Reading Time was significant in the Pre session z-ratio = 18.94, p_FDR_ < 0.001 (EMM = 0.98, SE = 0.05), in the Mid-session z-ratio = 10.64, p_FDR_ < 0.001 (EMM = 0.55, *p* < 0.001) and in the Post session z-ratio = 8.09, p_FDR_ < 0.001 (EMM = 0.42, SE = 0.05). Furthermore, the slope in the Pre session was significantly higher with respect to the Mid-session z-ratio = 9.31, p_FDR_ < 0.001 and to the Post session z-ratio = 11.95, p_FDR_ < 0.001. The slope in the Mid-session was also higher than the one of the Post session z-ratio = 2.79, p_FDR_ = 0.005. These data show that the influence of Initial Reading Time on self-paced reading time (in Pre, Mid and Post session) is larger at the start of the experiment, becoming progressively smaller in the mid and the post sessions.

Importantly, the same test showed an interaction between Session and Experiment *F*(4, 6238.9) = 5.16, *p* < 0.001. In accordance with the previous within-experiment analyses, post-hoc tests showed that in Exp. 1 Letter Reading Time diminished from Pre to Mid-session z-ratio = 2.45, p_FDR_ < 0.001 (EMM = 14.39, SE = 1.98), from Mid to Post z-ratio = 4.85, p_FDR_ = 0.021 (EMM = 4.85, SE = 1.98) and from Pre to Post z-ratio = 9.60, *p* < 0.001 (EMM = 19.25, SE = 2.00). In Exp. 2, Letter Reading Time diminished from Pre to Mid-session z-ratio = 8.68, p_FDR_ < 0.001 (EMM = 14.66, SE = 1.69) and from Pre to Post z-ratio = 8.16, p < 0.001 (EMM = 13.84, SE = 1.70) but not from Mid to Post (*p* = 0.63). Similarly, in Exp. 3, Letter reading time diminished from Pre to Mid z-ratio = 5.60, p_FDR_ < 0.001 (EMM = 10.03, SE = 1.79) and from Pre to Post z-ratio = 4.74, *p* < 0.001 (EMM = 8.52, SE = 1.80) but not from Mid to Post (*p* = 0.43) (see Figure 3C). Furthermore the Δ letter reading time (i.e., pre - post) were larger in Exp. 1 (EMM = 19.26, SE = 2.00) with respect to Exp. 2 (EMM = 13.85, SE = 1.70) z-ratio = 5.40, *p* = 0.047, and Exp. 3 (EMM =8.52, SE = 1.80) z-ratio = 3.99, *p* = 0.001 but also larger in Exp. 2 with respect to Exp. 3 z-ratio = 2.15, *p* = 0.041. These results show that even when using the non-accellerated reading a significant RAE is observed however, RAE is larger when reading acceleration is used with respect to the non-accelerated procedure (see Figure 3D).

Main effects of Session *F*(2, 6,257) = 127.89, *p* < 0.001, Initial Reading Time *F*(1, 83.90) = 232.70, *p* < 0.001 were not further analysed considering their involvement in more complex interaction effects explored above.

To summarize, these results are consistent with the correlation results, showing that the letter reading time observed across the different experimental sessions depends on participants’ initial letter reading time. By taking into account the initial reading time as a covarying predictor, a notable advantage provided by the reading acceleration procedure emerged. In fact, although an improvement in reading performance was found even when using the non-accelerated reading procedure (Exp. 3), the magnitude of the improvement was higher when reading acceleration was implemented (Exp. 1 and 2).

## 4 Discussion

Building on earlier research revealing the effectiveness of externally imposed reading rates by means of text disappearance (Breznitz et al., 2013), we developed a single-session computerized reading acceleration procedure for adult Italian readers. Previous studies on RAE have described the reading material merely as comprising short declarative sentences (Breznitz and Share, 1992; Breznitz et al., 1994, 2013; Breznitz, 1997a,b,c), with poor description of the necessary information required to replicate the effect, given that the actual characteristics of reading stimuli remain largely unknown. Therefore, we created a database of stimuli *ex-novo*, taking into account important linguistic characteristics such as sentence length, word frequency, and verb tenses. We administered the computerized reading acceleration task to typical adult readers with varying levels of reading abilities.

In the first experiment, during fast-paced reading conditions, two different types of text fading were used: continuous fading (a background-colored occlusor progressively covering the sentences) and letter-by-letter fading (character-wise erasure). Possible transfer effects of the two fading techniques on non-accelerated reading were also investigated by comparing self-paced reading times recorded before and after each fast-paced condition. Participants read faster during fading manipulations (~27 ms per letter during continuous fading, and ~ 39 ms per letter during letter-by-letter fading) relative to when no manipulation occurred (self-paced reading pre: ~62 ms per letter; self-paced reading post: ~55 ms per letter). This evidence aligns with the only previous RAE study on unimpaired adult English readers (Breznitz et al., 1994) which, using a word-wise fading procedure, reported that during accelerated conditions participants were able to read 12% faster than they normally could. Notably, in the present study an increase in reading speed of approximately 40% was observed in the fast-paced trials as compared to self-paced trials. Such marked difference between Breznitz et al. (1994) and the present results could be attributed to a number of factors, such as the sample size (*N* = 15 vs. *N* = 30), the reading stimuli used (passages vs. short sentences), the type of fading used (whole words vs. characters), the number of trials used for the fading manipulation (17 vs. 100) or during self-paced reading (17 vs. 25). However, the limited data reported in the previous study does not allow to make direct comparisons between the two studies (Breznitz et al., 1994). The fact that significantly shorter letter disappearing rates were achieved during continuous fading as compared to letter-by-letter fading (~ 27 ms vs. ~ 39 ms, respectively) is a relevant result for the future implementation of RAE and RAP protocols, especially when using common 60 Hz monitor; for faster monitor refresh rates these procedures might be virtually equivalent, although our study was not specifically designed to test this aspect.

Comparing results of Experiment 1 with similar studies in the literature, it is worth noting that in most previous RAE investigations (Breznitz and Share, 1992; Breznitz, 1997a,b,c) no differences were found in self-paced reading time evaluated before and after the acceleration procedure, while our results revealed a reduced letter reading time during the self-paced trials following the acceleration procedure. More specifically, participants had a letter reading time of ~62 ms in self-paced trials before the acceleration, whereas a mean reading time of ~55 ms was observed during self-paced trials after the acceleration, regardless of the specific fading manipulation employed. This reduction in letter reading time of approximately 10% is consistent with previous evidence from Bar-Kochva and Hasselhorn (2015) on German children, who reported an increase in reading speed, albeit associated with a decrease in comprehension levels, in self-paced post reading conditions as compared to self-paced pre ones (from 13 to 16 letters per second), regardless of the specific unit faded (whole words, single letters, or orthographic units) (Bar-Kochva and Hasselhorn, 2015). Our study and that of Bar-Kochva and Hasselhorn (2015) differ from previous RAE investigations mainly in terms of session duration and number of trials. In particular, both studies included more than one fast-paced reading block and more than two self-paced conditions. In most previous studies the number of sentences ranged from 6 to 20, while in Bar-Kochva and Hasselhorn’s study there were 22 in both self-and fast-paced blocks; in the present study we had 25 trials in the self-and 100 trials in the fast-paced blocks. Considering the cumulative effects of repeated exposure to reading acceleration previously reported by RAP studies (Korinth and Nagler, 2021), the long-lasting gain in reading fluency found in the two studies may be explained by the presentation of several fast-paced blocks. Thus, the neuroplastic mechanisms underlying RAP benefits may have been enabled by prolonged reading acceleration, albeit in a single session. Although Bar-Kochva and Hasselhorn (2015) did not find any differences in fast-paced reading times among the three groups of children who underwent different fading techniques, when investigating online effects of our manipulations we noticed that shorter letter disappearing rates were achieved during continuous fading as compared to letter-by-letter fading (~ 27 ms vs. ~ 39 ms, respectively). However, the two fading techniques did not differ in the transfer effects yielded in self-paced post reading conditions, as they both induced a similar reading speed enhancement; it may be that extending the duration of the experimental session or including more sessions could potentially facilitate the observation of such effects, if present.

In the second experiment, in addition to replicating the results of Experiment 1 in an independent sample of 30 unimpaired adult readers, we examined whether reducing visual crowding could improve the reading gain observed during RAE, by potentially inducing more accurate letter-string segregation and discrimination. To this aim, during fast-paced conditions, we manipulated the inter-letter spacing (i.e., standard vs. large) and tested potential transfer effects on self-paced reading blocks. Results confirm the main results of Experiment 1, showing an increased reading speed during self-paced reading blocks following reading acceleration, with an average letter reading time of ~64 ms, as compared to letter reading time in self-paced blocks preceding reading acceleration of ~70 ms, regardless of the specific inter-letter spacing employed. Contrary to our expectations, no significant effects of inter-letter spacing were found neither in the fast-paced nor in the self-paced reading conditions. Evidence from previous literature is mixed regarding the effects of inter-letter spacing on reading performance. While some studies have reported facilitatory effects on reading performance with larger inter-letter spacing (Spinelli et al., 2002; Perea et al., 2012, 2016; Zorzi et al., 2012; Scaltritti et al., 2019), others have reported inhibitory effects (Van Den Boer and Hakvoort, 2015; Galliussi et al., 2020; Korinth et al., 2020). Our results showed that inter-letter spacing does not affect the RAE in adult participants with unimpaired reading abilities. One potential explanation for the lack of such an effect might rely on participants’ reading abilities. Most previous studies have investigated the effects of inter-letter spacing on reading performance and word recognition in dyslexic readers, as visual crowding has been reported to be one of the core deficits of dyslexia (Martelli et al., 2009; Gori and Facoetti, 2015; Bertoni et al., 2019). Here, instead, participants were young adults with no reading difficulties, and it is possible that crowding manipulation is not relevant for fastening their reading speed.

In Experiment 3, we specifically isolated the contribution of reading practice without forced acceleration. This control experiment helps us in interpreting changes in reading speed during and/or after the acceleration, because it is possible that the decrease in reading time may emerge also without acceleration. Thus, instead of presenting fast-paced trials we only presented self-paced trials in shorter and longer blocks to mimic the structure of the previous two experiments. Results showed a decrease in self-paced reading time also without acceleration, but a similar decrease was not observed when comparing reading time in longer self-paced blocks that participants performed as an alternative to accelerated reading.

Correlation analyses on the whole sample (*N* = 90) across the three experiments showed that slower readers exhibited a higher gain in letter reading speed after the reading acceleration procedure. This is coherent with previous studies showing higher benefits for individuals with impaired reading abilities as compared to typical readers (Breznitz, 1997a,c; Breznitz and Leikin, 2001). However, to our knowledge, no studies reported a linear relationship between reading abilities and RAE-induced improvements, neither in typical readers nor in individuals with reading difficulties in the context of single-session designs. The dependency of reading acceleration benefits on individual characteristics suggest that the possible improvement of reading abilities spans over a continuum. Given the scarcity of studies concerning reading rehabilitation/enhancement approaches in adulthood, this result opens the possibility of improving the design of reading acceleration procedures.

The results of the correlation led us to consider the role of individual variability in determining the magnitude of the RAE across the three experiments via linear mixed effects models. First, this analysis confirmed the results of the correlation, showing that the letter reading time measured across the different sessions depends on the initial letter reading time. Second and most importantly, only taking into account the initial reading time as a covarying predictor we were able to show the advantage provided by reading acceleration in determining the magnitude of the RAE in Exp. 1 & 2. Specifically, although a significant improvement in reading performance was found even when using the non-accelerated reading procedure (Exp. 3), the RAE was larger when reading acceleration was implemented. This suggests that reading practice alone is effective in incrementing self-paced reading speed in adult normal readers at least within a single session. These considerations strongly suggest that the magnitude of the RAE induced by reading acceleration *per se* is possibly smaller than what previous studies showed, advocating for a deeper investigation of the interplay between reading practice and acceleration. A limitation of the comprehensive analysis comparing the reading speed gain across Exp. 1, 2 and 3 consists in the suboptimal estimation of individual variation in the “Session” effect of the Mixed Model. The size of the dataset did not allow for an estimation of a “Session” random slope and its absence may result in an overestimation of fixed effects (Barr, 2013). Therefore, the estimated fixed effects may be smaller than they appear, calling for a cautious interpretation of this specific finding.

Overall, the evidence reported here supports the possibility of eliciting reading acceleration even in a single session in individuals with normal levels of reading abilities. These results will promote future studies that better investigate the different cognitive processes underlying the observed benefits in reading performance induced by the reading acceleration procedure, as well as the underlying neurophysiological mechanisms and potential ways to use neuromodulation to boost RAE and RAP outcomes. For instance, it has been suggested that by forcing readers to visually follow the letters as they are erased from the screen, the reading acceleration manipulation enables them to overcome the reliance on slow phonological encoding processes and, thereby, to process words quickly and in a more holistic manner (Breznitz and Share, 1992; Breznitz, 2006; Breznitz et al., 2013). Thus, reading acceleration might switch information processing from a sequential and slow phonological path to a more direct and rapid visual stream (Nagler et al., 2016). Moreover, it has been shown that reading acceleration improves attention span, reduces distractibility, and enhances working memory abilities (Breznitz, 1988, 1997c; Breznitz and Share, 1992). Lastly, there is evidence that reading acceleration also increases direct word retrieval from the mental lexicon (Breznitz, 1987). Given that concepts stored in the mental lexicon can be accessed or decoded via their phonological or visual representation (Coltheart et al., 1993), the time constraint imposed by fast-paced reading by enhancing rapid visuo-attentional decoding abilities makes semantic information more likely to be retrieved directly and efficiently, consequently benefiting reading speed and comprehension (Nagler et al., 2014, 2016). However, it is worth noting that while visual acuity in the parafoveal region is reduced compared to the foveal region, parafoveal information is not completely neglected (Rayner, 1975). It has been shown that readers use information at the right of the word currently under fixation to enable efficient reading proficiency (Rayner et al., 2006a). One interesting question for future studies is whether the advantages of RAE may also be due to an increased parafoveal benefit, since a more efficient parafoveal processing may develop in the right visual field as information to the left of fixation, which could have a potentially distracting or slowing influence, is progressively removed. While this hypothesis seems compelling, it remains speculative and needs to be supported by eye-tracker data specifically addressing oculomotor behavior and parafoveal preview benefits during self-paced and fast-paced reading conditions. Conducting further studies in the unimpaired adult population, using methods such as EEG and eye-tracking, will lead to a more comprehensive understanding of the underlying neurophysiological and neurocognitive mechanisms, and will shed light on their potential interaction. Such research will also enable more sophisticated investigations at the level of psycholinguistic, semantic, methodological, and psychophysical parameters that could potentially impact RAE and RAP outcomes. Finally, transcranial alternating current stimulation, in combination with behavioral paradigms, have recently been shown to be effective in ameliorating cognitive and perceptual functions both in healthy individuals, aging, and neuropsychiatric populations (Grover et al., 2023; Di Dona et al., 2024). Thus, it would be interesting to investigate whether the application of brain stimulation during reading acceleration would enhance its beneficial outcomes, resulting in more robust or enduring effects.

The current results, along with Franceschini’s RAP study on Italian dyslexic children (Franceschini et al., 2017), suggest potential applications of such procedures in Italian-speaking clinical populations. Since the current paradigm has been implemented in Psychopy, once refined and validated, it could easily be distributed online via Pavlovia,^2^ a web-based platform to run behavioral and psychophysical experiments. This approach will not only provide many potential new insights from an experimental point of view but, importantly, it would also enable a greater number of individuals to benefit from remote and/or home-based reading acceleration. Crucially, given the scarcity of reading rehabilitation approaches in adulthood, the fact that this effect was easily elicited in typical readers with normal levels of reading abilities, may help in improving training design and implementation for slow and impaired readers, not only those with dyslexia, but also in aging (Stine-Morrow et al., 1996; Rayner et al., 2006b; De Luca et al., 2017; Liu et al., 2017; Warrington et al., 2018), in individuals with acquired brain lesions, or in other disorders (Tabet et al., 2020; Pei and O’Brien, 2021; Ratiu et al., 2022).

To conclude, in two distinct RAE experiments and in an additional control experiment on adult Italian participants with varying levels of reading abilities, we: (i) effectively replicated Breznitz’s findings in an independent way, using a paradigm as similar as possible to the original one; (ii) extended previous evidence on RAE demonstrating that the type of fading procedures could impact the online effects yielded by the RAE, (iii) showed that slower readers are characterized by higher improvements in reading speed; (iv) showed that inter-letter spacing does not seem to affect the RAE in unimpaired readers; (v) showed that improvements in self-paced reading speed observable within a single session can be obtained also with non-accelerated reading, although to a lesser degree.

## Data availability statement

The raw data supporting the conclusions of this article will be made available by the authors, without undue reservation.

## Ethics statement

The studies involving humans were approved by Ethical Committee of San Raffaele Hospital. The studies were conducted in accordance with the local legislation and institutional requirements. The participants provided their written informed consent to participate in this study.

## Author contributions

DAZ: Data curation, Formal analysis, Investigation, Methodology, Visualization, Writing – original draft, Writing – review & editing. GDD: Data curation, Formal analysis, Investigation, Methodology, Validation, Visualization, Writing – original draft, Writing – review & editing. MB: Investigation, Methodology, Writing – review & editing. FDB: Data curation, Methodology, Writing – review & editing. LR: Conceptualization, Funding acquisition, Project administration, Resources, Supervision, Writing – original draft.

## Footnotes

^1^ https://osf.io/8hwut/?view_only=b02d213936424fa99fb99efe4b39e5c3

^2^ https://pavlovia.org
