# Supplementary material for: Enhancing reading speed: the reading acceleration effect in Italian adult readers
Source: Front Psychol. 2024 Jul 31;15:1394579. doi: 10.3389/fpsyg.2024.1394579 (PMC11322054; doi:10.3389/fpsyg.2024.1394579)
Supplement: Supplementary file 1 [file Table_1.PDF]

Supplementary Table S1: Word Frequency and Target Position

		**Word Frequency** Mean Zipf (SD)				**Target Position**				
		Sentence	*Target*	*Distractor*	*Fillers*	*Initial*	*Initial-Mid*	*Mid*	*Mid-Final*	*Final*
**Block**	**List**									
**Self-Paced (N = 25)**	*List 1*	4.06 (0.74)	2.33 (1.67)	1.94 (1.32)	2.37 (1.63)	0.16	0.12	0.44	0.04	0.24
	List 2	4.09 (0.49)	1.89 (1.44)	1.93 (1.64)	2.10 (1.66)	0.12	0.20	0.40	0.12	0.16
	List 3	4.15 (0.65)	2.52 (1.36)	2.59 (1.40)	2.70 (1.63)	0.12	0.32	0.20	0.16	0.20
	AVG	4.10 (0.60)	2.25 (1.57)	2.15 (1.52)	2.39 (1.63)	0.13	0.21	0.34	0.10	0.20
**Fast-Paced (N = 100)**	*List 1*	4.16 (0.48)	2.78 (1.62)	2.63 (1.58)	2.84 (1.57)	0.11	0.23	0.26	0.21	0.19
	List 2	4.25 (0.70)	2.75 (1.54)	2.49 (1.46)	3.02 (1.70)	0.17	0.22	0.29	0.09	0.23
	AVG	4.20 (0.62)	2.77 (1.57)	2.56 (1.52)	2.93 (1.63)	0.14	0.22	0.28	0.15	0.21

Supplementary Table S2: Mean Word Number and Mean Letter Number per sentence

		**Mean Word Number**<br>Mean(SD)	**Mean Letter Number**<br>Mean(SD)
**Block**	***List***		
**Self-Paced (N = 25)**	*List 1*	10.80 (1.03)	55.40 (6.81)
	List 2	10.60 (0.91)	54.80 (7.06)
	List 3	10.70 (0.85)	54.00 (7.33)
	AVG	10.70 (0.92)	54.74 (7.00)
**Fast-Paced (N = 100)**	*List 1*	10.70 (1.38)	55.70 (7.25)
	List 2	11.00 (1.06)	55.30 (7.18)
	AVG	10.85 (1.23)	55.53 (7.20)
